# Integrating deep phenotyping with genetic analysis: a comprehensive workflow for diagnosis and management of rare bone diseases

**DOI:** 10.1186/s13023-024-03367-8

**Published:** 2024-10-08

**Authors:** Guozhuang Li, Kexin Xu, Xiangjie Yin, Jianle Yang, Jihao Cai, Xinyu Yang, Qing Li, Jie Wang, Zhengye Zhao, Aoran Mahesahti, Ning Zhang, Terry Jianguo Zhang, Nan Wu

**Affiliations:** 1 Department of Orthopedic Surgery, Peking Union Medical College Hospital, Peking Union Medical College and Chinese Academy of Medical Sciences, No. 1 Shuaifuyuan, Beijing, 100730 China; 2 Beijing Key Laboratory for Genetic Research of Skeletal Deformity, Beijing, 100730 China; 3 Key Laboratory of Big Data for Spinal Deformities, Chinese Academy of Medical Sciences, Beijing, 100730 China; 4 State Key Laboratory of Complex Severe and Rare Diseases, Peking Union Medical College Hospital, Peking Union Medical College and Chinese Academy of Medical Sciences, Beijing, 100730 China

**Keywords:** Deep phenotyping, Rare bone diseases, Precision medicine, Human Phenotype Ontology (HPO), Genetic testing, Genetic counseling, Multidisciplinary team (MDT)

## Abstract

Phenotypes play a fundamental role in medical genetics, serving as external manifestations of underlying genotypes. Deep phenotyping, a cornerstone of precision medicine, involves precise multi-system phenotype assessments, facilitating disease subtyping and genetic understanding. Despite their significance, the field lacks standardized protocols for accurate phenotype evaluation, hindering clinical comprehension and research comparability. We present a comprehensive workflow of deep phenotyping for rare bone diseases from the Genetics Clinic of Skeletal Deformity at Peking Union Medical College Hospital. Our workflow integrates referral, informed consent, and detailed phenotype evaluation through HPO standards, capturing nuanced phenotypic characteristics using clinical examinations, questionnaires, and multimedia documentation. Genetic testing and counseling follow, based on deep phenotyping results, ensuring personalized interventions. Multidisciplinary team consultations facilitate comprehensive patient care and clinical guideline development. Regular follow-up visits emphasize dynamic phenotype reassessment, ensuring treatment strategies remain responsive to evolving patient needs. In conclusion, this study highlights the importance of deep phenotyping in rare bone diseases, offering a standardized framework for phenotype evaluation, genetic analysis, and multidisciplinary intervention. By enhancing clinical care and research outcomes, this approach contributes to the advancement of precision medicine in the field of medical genetics.

**Supplementary Information:**

The online version contains supplementary material available at 10.1186/s13023-024-03367-8.

## Background

In the field of medical genetics, phenotypes hold a pivotal role, extending from the diverse pea plants cultivated by Mendel to the white-eyed fruit flies unexpectedly discovered by Morgan, to the replication of various human genetic variants in mice through gene editing technologies. Phenotypes serve as the external manifestations of the underlying genotypes, thereby constituting both a primary and crucial subject of genetic research. The adage "Phenotype is king, genotype is queen" underscores the principle that without a clear understanding of the phenotype, searching for a genotype is futile. This highlights the importance of phenotype in guiding genetic research and analysis [1, 2].

With advancements in next-generation sequencing technologies (e.g., Exome sequencing, ES, Genome sequencing, GS) and bioinformatics analysis, our capacity to detect genomic variants has significantly increased. For the interpretation of these variants, the American College of Medical Genetics and Genomics (ACMG) regularly publishes and updates the standards and guidelines for the interpretation of sequence variants, which have become the global standard for decoding genetic variants [3]. These technologies and guidelines underscore a phenotype-driven strategy for interpreting genomic data.

Despite the undeniable importance of phenotype in the domain of medical genetics, the field lacks detailed guiding protocols and standards for phenotype evaluation. Current clinical records and medical literature often provide imprecise and incomplete descriptions of phenotypes [4]. For instance, reports might state that a patient has scoliosis without specifying the age of onset (childhood? adolescence? or adulthood?), the etiology (congenital vertebral malformation? degenerative changes? neuromuscular conditions? or idiopathic?), or the severity of the curvature (measured in Cobb angle? classified by the Winter's classification or the Lenke classification?). Such vagueness hampers our ability to fully comprehend the patient's condition and complicates the comparison of research outcomes across different study centers.

Therefore, this article is composed to elucidate the practice of integrating deep phenotyping with genetic analysis, a comprehensive workflow for diagnosis and management of rare bone diseases in China, provided by the Genetics Clinic of Skeletal Deformity at Peking Union Medical College Hospital (PUMCH).

## Main text

### Deep phenotyping and genetic analysis in the Genetics Clinic of Skeletal Deformity at PUMCH

**Fig. 1:**
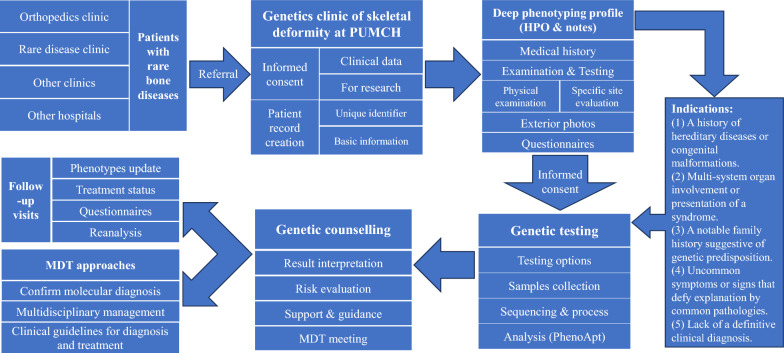
Workflow for diagnosis and management of rare bone diseases at the Genetics Clinic of Skeletal Deformity of PUMCH

Deep phenotyping represents a precise and comprehensive methodology employed for the detailed analysis and assessment of patients' multi-system phenotypes. This approach encompasses the evaluation of symptoms, signs, a wide array of medical examination and laboratory results, along with other medically relevant information. In the realm of clinical diagnosis and medical research pertaining to rare bone diseases, deep phenotyping emerges as a crucial element. The advent of precision medicine is founded upon three principal dimensions: deep phenotyping, stratified medicine, and targeted therapy. Among these, deep phenotyping lays the groundwork for both stratified medicine and targeted therapy [5]. The department of orthopedic surgery from PUMCH established China's first Genetics Clinic of Skeletal Deformity in 2019. Through extensive practice and accumulation of experience, a comprehensive workflow for diagnosis and management of rare bone diseases was developed, which encompasses a sequence of processes: routine outpatient screening and referral, deep phenotyping at the Genetics Clinic of Skeletal Deformity, genetic testing, analysis of sequencing data, multidisciplinary consultations, and regular follow-ups (Fig. 1).

### Referral, informed consent, and profile

Patients may be referred to the Genetics Clinic of Skeletal Deformity from various clinics within PUMCH, such as the orthopedics clinic, rare disease clinic, and others, or from external hospitals.

For patients referred to the Genetics Clinic of Skeletal Deformity, the initial step involves obtaining informed consent. This process informs patients about the necessity of collecting various clinical information/data, the principles of confidentiality, and the potential for research use, with all studies approved by the ethics committee of PUMCH. Upon obtaining informed consent, the second step involves clinicians or medical students creating a comprehensive patient profile. This process includes assigning a unique identifier to each patient within a multimodal database, completing basic information, and constructing family pedigrees.

### Deep phenotyping in deep phenotyping profile by HPO terms

The third and most crucial step involves deep phenotyping. The basis for the deep phenotyping is the deep phenotyping profile used by the Genetics Clinic of Skeletal Deformity. The deep phenotyping profile is a structured checklist, aligned with the standard HPO catalogue [6], enriched by additional notes (Supplementary material). All identified phenotypic abnormalities are recorded using the standard HPO terms, with detailed textual descriptions provided for complex phenotypes not covered by the HPO. The deep phenotyping profile encompasses three main aspects:

1. Deep phenotyping profile is structured with the current patient's HPO terms at the forefront, presenting all phenotypic information utilizing HPO. In the digital deep phenotyping profile, a copy button is provided to swiftly duplicate all patient HPO terms.
2. Basic details about the patient's parents, including their ages and occupations at the time of the patient's birth, and whether there was consanguineous marriage. The patient's perinatal conditions, including the health status and the exposure to toxic or harmful environmental factors of the pregnant mother, prenatal screening results, and the newborn's condition at birth. Patient growth parameters are also accurately documented.
3. A systematic review of systems, where each system is thoroughly examined through targeted questioning and necessary physical examinations, including oral inspections, muscle strength tests, neurological reflex examinations, and tests for joint hypermobility, with reference to the patient’s completed medical examinations and testing.

For phenotypes that cannot be assessed through interviews and physical examinations, such as arrhythmia, rheumatoid arthritis, and neurodevelopmental issues, patients are referred to the appropriate specialist departments for further assessment. These evaluations are tracked and recorded in the patient’s profile.

Additionally, the clinic captures photographs/videos of distinctive phenotypic features, such as the razorback deformity in patients with scoliosis or excessive joint mobility in patients with Ehlers-Danlos syndrome. Patients also complete relevant questionnaires to assess their subjective experiences, including the 36-Item Short Form Survey (SF-36) [7], the Oswestry Disability Index (ODI) [8], and the Scoliosis Research Society Score-22 (SRS-22) for patients with scoliosis [9], among others.

### Genetic testing, analysis, and counseling

Based on the results of deep phenotyping, the team at the Genetics Clinic of Skeletal Deformity will advise whether patients require genetic testing based on the following five clear indications:

1. A history of hereditary diseases or congenital malformations.
2. Multi-system organ involvement or presentation of a syndrome.
3. A notable family history suggestive of genetic predisposition.
4. Uncommon symptoms or signs that defy explanation by common pathologies.
5. Lack of a definitive clinical diagnosis.

For those recommended for genetic testing, a suggested method of genetic analysis is provided. Currently, the mainstream genetic testing approach at the clinic involves trio-based GS combined with RNA sequencing. Upon patient consent and the signing of the informed consent form for genetic testing, samples from the patient and their family are collected for subsequent sequencing and interpretation of genetic data. In genetic analysis, we integrated PhenoApt—a machine-learning-based tool for phenotype-driven gene prioritization [10]. Utilizing PhenoApt, we leveraged extensive phenotypic data from deep phenotyping to bolster genetic analysis and diagnostics.

Following the genetic testing results, patients undergo genetic counseling, which includes interpretation of the genetic report, assessment of genetic risks for the patient and their family, and provision of support and guidance for diagnosis and treatment. For patients requiring multidisciplinary consultation, the team organizes and participates in MDT meetings to provide comprehensive management for patients with challenging rare bone diseases or genetic variants.

### MDT approaches

Utilizing MDT approaches, the Genetics Clinic of Skeletal Deformity team at PUMCH has confirmed molecular diagnoses for over a hundred patients. Through focused discussions among relevant departments, the MDT has provided comprehensive multidisciplinary management for patients with challenging rare bone diseases, those presenting skeletal abnormalities, or carrying genetic variants. By amalgamating MDT clinical experiences and consulting specialists across departments, the team is committed to formulating evidence-based clinical guidelines for the diagnosis and management of specific rare bone diseases. In 2023, the team spearheaded the development and publication of China's first clinical guidelines for diagnosis and treatment of the Ehlers-Danlos syndromes [11], which will shorten the diagnostic odyssey and solve the unmet medical needs of the patients.

### Follow-up visits

Regular follow-up visits are crucial for patients with rare bone diseases, irrespective of their treatment approach—conservative or surgical—due to the potential evolution of phenotypes over time. The clinic underscores the significance of dynamic deep phenotyping at each follow-up. This comprehensive patient care cycle includes updating phenotypic information, monitoring treatment progress, administering tailored questionnaires for deeper insights, and reanalyzing genetic data for patients who initially tested negative. Such reanalysis is informed by emerging new phenotypes and the latest research findings, ensuring a more informed and responsive treatment strategy [12].

### Case of 16p11.2 microdeletion syndrome in a pediatric patient

**Fig. 2:**
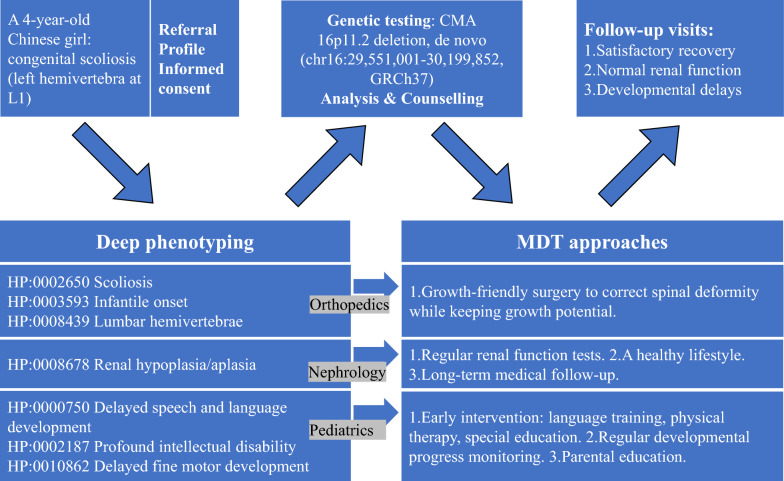
A pediatric case of 16p11.2 microdeletion syndrome illustrating our comprehensive workflow for the diagnosis and management of rare bone diseases

We present a pediatric case of 16p11.2 microdeletion syndrome, accompanied by a schematic diagram (Fig. 2), to illustrate our comprehensive workflow for the diagnosis and management of rare bone diseases:

A 4-year-old Chinese girl from the first pregnancy of a non-consanguineous healthy young couple was diagnosed with congenital scoliosis (left-sided hemivertebra at L1) in the orthopedic clinic at PUMCH. She was referred to the Genetics Clinic of Skeletal Deformity. After obtaining informed consent, the patient profile was created, and deep phenotyping was conducted. The phenotypic assessment included a full spine X-ray and detailed medical history, revealing:

HP:0002650 Scoliosis

HP:0003593 Infantile onset

HP:0008439 Lumbar hemivertebrae

Renal ultrasound indicated renal hypoplasia, with the patient's kidney size measuring 2 standard deviations below the mean for age and sex [13], corresponding to:

HP:0008678 Renal hypoplasia/aplasia

Given the suspicion of developmental delays, a comprehensive pediatric assessment was conducted, identifying:

HP:0000750 Delayed speech and language development

HP:0002187 Profound intellectual disability

HP:0010862 Delayed fine motor development

These findings raised the suspicion of a 16p11.2 microdeletion syndrome [14]. Following informed consent for chromosomal microarray analysis (CMA), results confirmed a de novo 16p11.2 deletion (chr16:29,551,001–30,199,852, GRCh37). Genetic counseling was provided, and an MDT including orthopedics, pediatrics, and nephrology was assembled. The orthopedic plan included a growth-friendly surgery to correct spinal deformity while preserving growth potential. The pediatric team recommended early intervention, including language and communication training, physical therapy, occupational therapy, and special education. Regular developmental progress monitoring and parental education were emphasized. The nephrology team advised regular renal function tests, a healthy lifestyle, and long-term medical follow-up.

Annual follow-ups at the Genetics Clinic of Skeletal Deformity were scheduled. The final follow-up results revealed the patient showed good postoperative recovery from spinal surgery, with developmental delays persisting but normal renal function.

This case highlights the importance and the utility of deep phenotyping and the workflow of the Genetics Clinic of Skeletal Deformity at PUMCH in diagnosing rare bone diseases. The MDT approach was crucial for comprehensive management and family support.

## Conclusions

Deep phenotyping for rare bone diseases holds significant implications for precision medicine, providing a standardized framework for comprehensive phenotype evaluation, genetic analysis, and personalized intervention. Deep phenotyping enables stratification of patients into subgroups; for instance, those with the 16p11.2 deletion are classified into neurodevelopmental, urinary, reproductive, and musculoskeletal categories [14]. Employing deep phenotyping within our workflow yields comprehensive phenotypic data that significantly advances both disease understanding and genetic interpretation. This approach facilitates the identification of phenotypic patterns and the development of the predictive scoring system [15], the expansion of phenotypic spectra [16], and a deeper insight into anomalies associated with congenital deformities [17]. Previous research has established that deep phenotyping links *TBX6* variants to congenital scoliosis [18], renal dysplasia [13], and Mayer-Rokitansky- Küster-Hauser syndrome [19], thereby enabling refined analyses of genotype–phenotype interactions. Moreover, by integrating PhenoApt with standardized HPOs from deep phenotyping, our workflow accelerates genetic analysis and diagnosis through precise prediction of pathogenic genes [10].

The deep phenotyping in our workflow is valuable but not without its challenges and limitations. Subjectivity is notable, as deep phenotyping is dependent on the evaluator's understanding of abnormal phenotypes. For example, neglecting the assessment of oral anomalies can lead to the frequent omission of conditions such as high palate and ankyloglossia. The use of a checklist-embedded deep phenotyping profile is therefore crucial to mitigate this issue (Supplementary material). Additionally, the manual selection or entry of HPO terms by clinicians or genetic counselors is a time-consuming process, which can hinder the efficiency of deep phenotyping. The development and application of artificial intelligence-driven software that automatically extracts HPO terms from electronic medical records represent a significant adjunct to enhancing the efficiency of deep phenotyping [20]. Furthermore, obtaining informed consent is essential before initiating deep phenotyping, given its comprehensive and detailed nature, which is both time-intensive and requires a high level of patient compliance for periodic updates. Effective communication and thorough informed consent are paramount to ensure patient understanding and engagement in the deep phenotyping process.

By systematically cataloging phenotypic data and integrating multidisciplinary approaches, deep phenotyping facilitates precise diagnosis, tailored treatment strategies, and the development of clinical guidelines. Through its role in elucidating disease mechanisms and informing therapeutic interventions, deep phenotyping for rare bone diseases represents a pivotal step towards improving patient outcomes and advancing medical genetics research globally.

## Supplementary Information

Additional file1

## Data Availability

Not applicable.

## Abbreviations

HPO: Human Phenotype Ontology
PUMCH: Peking Union Medical College Hospital
MDT: Multidisciplinary team
ES: Exome sequencing
GS: Genome sequencing
ACMG: American College of Medical Genetics and Genomics
SF-36: 36-Item Short Form Survey
ODI: Oswestry Disability Index
SRS-22: Scoliosis Research Society Score-22
