# Supplementary material for: Integrating deep phenotyping with genetic analysis: a comprehensive workflow for diagnosis and management of rare bone diseases
Source: Orphanet J Rare Dis. 2024 Oct 8;19:371. doi: 10.1186/s13023-024-03367-8 (PMC11462960; doi:10.1186/s13023-024-03367-8)
Supplement: Supplementary file 1 — Additional file1 [file 13023_2024_3367_MOESM1_ESM.pdf]

Deep Phenotyping Profile

HPO of current patient: Copy

HP:0002650 Scoliosis 脊柱侧弯 ×

Basic information

Patient's father's age at birth ______

Patient's father's job at birth ______

Patient's mother's age at birth ______

Patient's mother's job at birth ______

Clinical and Sample Information

Age of onset:

______

Major medical history:

______

Laboratory test records and results:

______

Family history:

______

Clinical information

Prenatal and perinatal period

☐ Unchecked ☐ Normal ☐ Intrauterine growth retardation IUGR ☐ Oligohydramnios ☐ Polyhydramnios ______ Week
☐ Cystic water tumor/NT thickening ☐ Fecal dye ☐ Umbilical cord around neck
☐ Myocardial echo focus ☐ Intestinal echo
☐ Developmental arrest ☐ Meningocele ☐ Increased transparency on the back of the neck ☐ Congenital diaphragmatic hernia

Notes:

______

Mother's pregnancy

G ______

P ______

Number of miscarriages ______

Reason

☐ Abortion ☐ Unknown ______ Week

Pregnancy

☐ Take medicine (eg. antiepileptic drugs) ______

☐ Exposure to drugs (eg. CO) ______

☐ Sick (eg. DM) ______

☐ Smoking ☐ Severe pregnancy reaction ☐ Not taking folic acid ☐ High temperature exposure ☐ Other ______

Notes:

______

Deep Phenotyping Profile

Birth condition

- [ ] GA at birth ____________ Fetal
- [ ] Prematurity ____________ (Which week)
- [ ] Birth weight ____________
- [ ] Vaginal birth - [ ] Caesarian section - [ ] Dystocia - [ ] Midwifery - [ ] Fetal distress - [ ] Neonatal hypotonia
- [ ] Neonatal respiratory distress - [ ] Ectopic ossification - [ ] Others ____________

Notes:

Age of onset

- [ ] Unknown - [ ] Congenital onset - [ ] Embryonal onset - [ ] Fetal onset - [ ] Neonatal onset - [ ] Infantile onset - [ ] Childhood onset
- [ ] Juvenile onset - [ ] Adult onset - [ ] Middle age onset - [ ] Late onset

Notes:

Growth parameters (percentage)

Weight ____________

Height ____________

Head circumference ____________

Measurement age ____________

Menarche age ____________

Notes:

Motor/Cognitive development

- [ ] Normal - [ ] Delayed fine motor development - [ ] Delayed gross motor development - [ ] Delayed speech and language development
- [ ] Intellectual disability - [ ] Learning Disability - [ ] Developmental regression

Notes:

Eye defect and vision

- [ ] Normal - [ ] Hypertelorism - [ ] Proptosis - [ ] Blue sclerae - [ ] Long palpebral fissure - [ ] Abnormal size of the palpebral fissures
- [ ] Ectropion - [ ] Cataract - [ ] Strabismus - [ ] Anophthalmia - [ ] Eye defect - [ ] Aniridia - [ ] Microphthalmia - [ ] Ophthalmoplegia
- [ ] Blindness - [ ] Optic atrophy - [ ] Rod-cone dystrophy - [ ] Ptosis

Notes:

Ear defect and hearing

- [ ] Normal - [ ] Sensorineural hearing impairment - [ ] Conductive hearing impairment - [ ] Mixed hearing impairment - [ ] Ear pit
- [ ] Cupped ear - [ ] Satyr ear - [ ] Amino sugar II-induced ototoxicity - [ ] Abnormality of the outer ear - [ ] Low-set ears - [ ] Protruding ear
- [ ] Attached ears - [ ] Otitis media - [ ] Hearing impairment

Notes:

Nose

- [ ] Normal - [ ] Wide nasal base - [ ] Slender nose - [ ] Hook nose - [ ] Bulbous nose - [ ] Shallow nasolabial fold - [ ] Anteverted nares

Notes:

Deep Phenotyping Profile

Mouth

- [ ] Normal - [ ] Oral cleft - [ ] High palate - [ ] Narrow mouth - [ ] Thin vermilion border - [ ] Multi-lace - [ ] Optic atrophy
- [ ] Abnormality of the dentition

Notes:

____

Craniofacial

- [ ] Normal - [ ] Macrocephaly - [ ] Microcephaly - [ ] Abnormality of the philtrum - [ ] Small jaw - [ ] Mandibular protrusion
- [ ] Prominent forehead - [ ] Hyperplasia of midface - [ ] Abnormal oral cavity morphology

Notes:

____

Behavioral characteristics

- [ ] Normal - [ ] Autistic behavior - [ ] Autism traits - [ ] Coercive barriers - [ ] Stereotypy - [ ] Personality disorder - [ ] Schizophrenia
- [ ] Other psychiatric symptoms _

Notes:

____

Skin and hair

- [ ] Normal - [ ] Abnormal hair - [ ] Abnormality of the nail - [ ] Abnormality of connective tissue - [ ] Water scar - [ ] Ichthyosis
- [ ] Hyperpigmentation of the skin - [ ] Hypopigmentation of the skin - [ ] Cafe-au-lait spots - [ ] Foliar hypopigmentation
- [ ] Neoplasm of the skin - [ ] Alopecia - [ ] Low posterior hairline - [ ] Skin rash - [ ] Trichorrhexis nodosa - [ ] Capillary hemangioma
- [ ] Abnormal blistering of the skin - [ ] Lumbosacral clumps of hair - [ ] Abnormal eyebrow morphology

Notes:

____

Respiratory system

- [ ] Normal - [ ] Respiratory failure - [ ] Apnea - [ ] Poor ventilation - [ ] Hyperventilation - [ ] Recurrent respiratory infections - [ ] Dyspnea
- [ ] Recurrent pneumonia - [ ] Pectus excavatum - [ ] Pectus carinatum - [ ] Thoracic collapse - [ ] Bilateral asymmetry

Notes:

____

lung

- [ ] Unchecked - [ ] Normal - [ ] Pulmonary obstruction - [ ] Restrictive ventilatory defect - [ ] Mixed ventilatory dysfunction
- [ ] Normal diffusing function - [ ] Decreased DLCO

Notes:

____

Cardiovascular

- [ ] Normal - [ ] Cardiomyopathy - [ ] Hypertrophic cardiomyopathy - [ ] Arrhythmogenic right ventricular cardiomyopathy ARVC
- [ ] Arrhythmia - [ ] Aortic stenosis - [ ] Atrial septal defect - [ ] Hypoplastic left heart - [ ] Ventricular septal defect ______
- [ ] Dilated cardiomyopathy - [ ] Syncope - [ ] Patent foramen ovale - [ ] Abnormality of cardiac ventricle - [ ] Tetralogy of Fallot
- [ ] Bicuspid aortic valve - [ ] Pre-excitation syndrome - [ ] Aortic root dilation - [ ] Other structural myocardial abnormalities (please specify)

Notes:

____

Neck

- [ ] Normal - [ ] Short neck - [ ] Torticollis - [ ] Limitation of neck motion - [ ] Webbed neck - [ ] Other ______________

Notes:

____

Deep Phenotyping Profile

Neurological

- [ ] Normal
- [ ] Seizure
- [ ] Abnormality of movement
- [ ] Ataxia
- [ ] Feeling detached
- [ ] Abnormal of corpus callosum
- [ ] Ventricular dilatation
- [ ] Brain atrophy
- [ ] Abnormality of the cerebral white matter
- [ ] Periventricular nodule
- [ ] Intracranial mass

Notes:

Gastrointestinal

- [ ] Normal
- [ ] Abdominal wall defect
- [ ] Pyloric stenosis
- [ ] Tracheoesophageal fistula
- [ ] Gastroparesis
- [ ] Eosinophilic esophagitis
- [ ] Gastroesophageal reflux
- [ ] Chronic vomiting
- [ ] Chronic diarrhea
- [ ] Constipate
- [ ] Anal atresia
- [ ] Intestinal pseudo-obstruction
- [ ] Esophageal atresia
- [ ] Inflammation bowel disease
- [ ] Hepatic failure
- [ ] Splenomegaly
- [ ] Aganglionic megacolon
- [ ] Hepatomegaly
- [ ] Liver function test value increased
- [ ] Umbilical hernia
- [ ] Inguinal hernia
- [ ] Scrotal hernia

Notes:

Skeleton

- [ ] Normal
- [ ] Flexion contracture
- [ ] Abnormal foot morphology
- [ ] Polydactyly
- [ ] Syndactyly
- [ ] Abnormality of limbs
- [x] Scoliosis
- [ ] Hyperlordosis
- [ ] Kyphosis
- [ ] Scoliosis(side raised)
- [ ] Vertebral fusion
- [ ] Abnormal vertebral morphology
- [ ] Tall stature
- [ ] Short stature
- [ ] Overgrowth
- [ ] Joint hypermobility
- [ ] Recurrent joint dislocation
- [ ] Cubitus varus
- [ ] Cubitus valgus
- [ ] Antecubital pterygium
- [ ] Genu varum
- [ ] Genu valgum
- [ ] Cutaneous finger syndactyly
- [ ] Camptodactyly
- [ ] Which
- [ ] Recurrent fractures
- [ ] Limb undergrowth
- [ ] Bowing of the long bones
- [ ] Skeletal muscle atrophy
- [ ] Muscle weakness

Notes:

Genitourinary

- [ ] Normal
- [ ] Unknown gender of vulva
- [ ] Hypospadias
- [ ] Hydronephrosis
- [ ] Polycystic kidney dysplasia
- [ ] Abnormality of the bladder
- [ ] Renal cyst
- [ ] Renal hypoplasia/aplasia
- [ ] Renal insufficiency
- [ ] Nephrotic syndrome
- [ ] Tubular necrosis
- [ ] Partial duplication of kidney
- [ ] Horseshoe kidney
- [ ] Abnormality of the ureter
- [ ] Abnormality of the urinary
- [ ] Cryptorchidism
- [ ] Abnormality of the testis
- [ ] Polycystic ovary
- [ ] Bicornuate uterus
- [ ] Abnormal external genitalia
- [ ] Primordial uterus
- [ ] Double uterus
- [ ] Mediastinal uterus
- [ ] No vagina
- [ ] Vaginal atresia
- [ ] Oblique vaginal septum
- [ ] Transverse vaginal septum

Notes:

Endocrine System

- [ ] Normal
- [ ] Diabetes insipidus
- [ ] Abnormality of the adrenal glands
- [ ] Hypoparathyroidism
- [ ] Hypothyroidism
- [ ] Pheochromocytoma
- [ ] Exocrine pancreatic insufficiency
- [ ] Hyperthyroidism
- [ ] Paraganglioma
- [ ] Hyperparathyroidism
- [ ] Diabetes mellitus

Notes:

Metabolic

- [ ] Normal
- [ ] Hyperalaninemia
- [ ] Low plasma L-carnitine
- [ ] Hypoglycemia
- [ ] Organic aciduria
- [ ] Elevated serum creatine phosphokinase
- [ ] Hyperglycemia
- [ ] Ketosis
- [ ] Organic acidemia
- [ ] Hyperammonemia
- [ ] Lactic acidosis
- [ ] Aminoaciduria
- [ ] Unusual color smell
- [ ] Pyruvate-elevated aminoacidemia
- [ ] Hyponatremia

Notes:

Hematology

- [ ] Normal
- [ ] Immunodeficiency
- [ ] Anemia
- [ ] Elevated iron ions
- [ ] Neutropenia
- [ ] leukemia
- [ ] Bone marrow hypocellularity
- [ ] Thrombocytopenia
- [ ] Thrombus
- [ ] Pancytopenia
- [ ] Bleeding disorders
- [ ] Pure red cell aplasia

Notes:

Deep Phenotyping Profile

Other notes

- ☐ Recurrent fever ☐ Muscle biopsy ____________________
- ☐ Abnormal test indicators ____________________
- ☐ Specific medical history ____________________
